# How to Conduct a City Practice

**Published:** 1897-10

**Authors:** C. B. Blackmarr

**Affiliations:** Jackson, Mich.


### How to Conduct a City Practice.

BY C. B. BLACKMARR, D.D.S., JACKSON, MICH.

*Gentlemen of the Michigan Dental Association:*

I would have been glad to have written on this subject when I was about eighteen years of age, for then I thought I knew how to conduct a city practice or anything else; but later in life, and especially since the money panic of 1893, I have lost that confidence in myself. Detroit and Battle Creek are both rather ideal cities to me, and whenever I visit the dentists there and here, I find them out on the river or at the flats, eating cheese, onions, etc., a good deal, and fishing and drinking a little; so I'm not sure but that is the way to conduct a city practice.

Prof. Hoff has told you how to start a city practice, and I consider his subject covers nearly the whole ground, because a practice started well is more than half the battle won. A practitioner who has had education in college and elsewhere for his work, and who is well equipped, mentally and physically, for the practice he is to undertake, will usually have success, so long as he remains so.

I imagine that when our president asked me to write on this subject, he wished me to tell how, in my opinion, a young man should conduct himself and his practice in a city, from the time he graduated out of college, and all ready, according to Prof. Hoff, to start his practice. Now, I think I could sum up all my advice to this young man by quoting an old saying, "When in Rome, do as the Romans do," especially the good things the Romans do and avoiding the bad things they do. And again, "Anything that is worth doing at all is worth doing well." "Well enough," a practical acquaintance of mine used to add.

The great trouble with most of us all is that we are not sure enough just what is worth doing, nor sure just what we are trying to do. Cities have character the same as individuals, and what will please in one city, will not in another. Each city also has several distinct classes of people to please and deal with. And it seems to me that the young man must decide which city and which class of people he is best adapted to please. There are some things every city and all classes will appreciate, such as education, honesty, skill, tact, etc., but some classes will appreciate them more than others. A practitioner must certainly please his patients in order to succeed. It is almost impossible for one man to please all classes, one certain class wishing a certain grade of operations and treatment, while another class will want a perfectly-different usage; and it requires more than an ordinary man to adapt himself to several classes and please them all. It requires tact and skill enough in dentistry to please one class. It is enough to ask of one man, and I have seen several dentists pretty tired doing even that.

There is the aristocratic class, which as a class are of wealthy, occasionally snobbish, who desire to go nowhere their associates or peers do not. They enjoy exclusiveness. They show this at their hotels, clubs, homes, etc. They desire everything done for their personal comfort, enjoyment and gratification that they can afford, and the dentist who can, by his reputation, manner, appointments, etc., please this class, will usually be well paid for his extra time spent doing operations for them. And right here I want to be understood in that I do not believe in the young dentist joining every or any church, club, society organization, etc., simply because he imagines his patients or those he wishes for patients would like to have him join. He should join because he would himself enjoy the societies. He must not have a clean and nice office and appointments simply because he thinks his patients wish it, but because he himself enjoys them. He will then be natural, and act natural, and naturally please those whom he is best qualified to please. I know of a young man who it would seem had joined every organization known in the city simply to become better known and to increase his practice; but later it seemed as if every member knew of his only object in joining, and were accordingly disgusted. So I believe that if a man really thinks best to join, and enjoys and receives benefit from the associations, and if he can be one of the members "in spirit and in truth" he would join the club whether it be the "Shriners" or the "Salvation Army." Then again there is the society class to please; but a man must not go in society to try and please the society people. He must go to please himself, and unless he can be "one of them" he will be thought more of by them by remaining away from their gatherings.

Now, a man must not remain away from public or society meetings from sheer indifference. If he does he will have the same treatment from the public toward him.

Then there is the business or commercial class of patients who need an entirely different treatment to be pleased. All their dealings and habits are of dispatch and promptness, and they will expect the same treatment from their dentist in regard to appointments, operations, etc. Again, there is the manual laboring class, factory-help, etc., who want operations done for them at all times of day and night, Sundays not excepted, and done as quickly and cheaply as possible. Offices to please this class must not be too pretentious nor imposing, and must be arranged to do work with as little waste of time as possible.

The different class of operations to be done for the different classes in a city requires a good amount of judgment and tact on the part of the operator. And to decide whether to have a quiet, elegant, exclusive office, or one like some barber shops, and with a balcony on in front for the band to play on, has driven many a man to an attack of the "blues." It seems to me that a man must decide soon after commencing practice whether he is to conduct a general practice of dentistry or a specialty. You will notice that in a city. I believe in specialties. I think the tendency of the times demands it. It seems to me that specialists in the practice of medicine succeed best, such as surgery, eye and ear, nose and throat, nervous diseases, etc., etc.

There is another idea that I believe in, and which I think good advice to the young dentist, and that is this: to be interested in and to work at only one specialty or business, if any one might call it so. Do not try to make a success of both dentistry and some other business, like mining, real estate, etc., etc. I believe men who have succeeded best in this life are those who have made a specialty of some one thing. Now, however, if a practitioner happens to earn more money than himself and family can or do spend, I think he should invest this money in a place where he can have it in money at any time needed for himself in his practice or family. And also if a practitioner for recreation gets interested in farm, flowers, garden, animals, etc., let him spend the time and interest needed for the purpose of recreation outside of his office hours, and not let it interfere with his regular work. In conducting a city practice, the matter of advertising seems to interest some if not all of us, and it seems to me that each and every one advertises or makes known his presence and wishes in a city, in some way, from the family practitioner, who tells by office and appearance, and notices here and there the class he wants to work for, to the dentist who says in the daily papers that he will not hurt at all, and will make lovely artificial teeth for $5.00, upward and downward, a set.

These different methods of advertising to attract different classes, each one a matter of judgment and taste, makes me think of the story of two married men, one of whom was named Johnson, who foolishly went down town one evening to get drunk. Toward morning both came toward their homes, arm in arm. Upon arriving at the home of Johnson they hollooed until the good wife of Johnson raised the window and asked who was there, and they both replied "It's me and Johnson, come down and pick out Johnson." His father-in-law met him at the door and said to him sadly, "Drunk again." "Whist," says Johnson, "So am I."

DISCUSSION.

Dr. Metcalf: I don't know that I can say anything in regard to starting a practice except as Horace Greeley said, "The way to resume is to resume," and that is the way to start a practice. The principal thing is suitable preparation, which can only be had by a thorough course in one of our well-equipped colleges. With that to start on, one will certainly have a foundation that will carry him to success. After the preparation and the office opened, the first thing on the part of a dentist is cleanliness. It is one of the most essential things a dentist has to attend to, not only in regard to himself personally, not only to wash his hands somewhere outside, but to wash his hands in a place near enough so that the next patient will know of it. The next thing necessary is clean napkins. I have had many a patient who came to me, not because I was a good dentist, but because they observed I always used a clean napkin. Take a sensitive patient and place a soiled napkin to their mouth, why, it is enough to turn the stomach of a crocodile. A clean mouth is a pretty good thing. I don't think that a dentist during his office hours ought to indulge in the use of tobacco or anything that will give an unpleasant odor to the breath. I do not say anything as to after office hours. And as to those stronger beverages, leave them to us older ones, like Dr. Field, Dr Taft and myself. Another essential to every practitioner is that of courtesy. I don't know of anything that does as much good as courtesy.

These little things I have mentioned will not only insure a good practice, but there goes with it a good reputation, and with economy, a sure competency to every dentist. If there is any one thing that I regret, it is that I have been obliged to leave my profession. It is the noblest profession in the world. When I began, our instruments were of the crudest kind. With the exception of forceps, we made our own. The old-fashioned excavators we had to shape and form and temper for ourselves. Now with the finest, most delicately made instruments in the hands of a competent man, how much can be accomplished.

Dr. Cleveland: Those who have preceeded me have taken the work off my shoulders. You will sometimes see men well prepared in an educational way, but they don't seem to get along. They lack tact. I think tact and kindness are two things, which if they are not natural, we ought to educate ourselves in this direction, if possible.

Dr. Essig: One of the principle things in my mind, is fulfilling your engagements promptly. Also doing the best work in your power. Another thing don't keep a patient waiting in the chair with rubber dam in the mouth. Though you may do the best of work, they will feel that you have neglected them.

Dr. Taft: A young man who crowds himself into a dental college, whether prepared or not, and works himself through the three courses, cannot feel that he is fully equipped in a preparatory way for his profession, while the fact is, that perhaps not one-half of the young men who graduate from our college are as well equipped as they ought to be, and might be, if they would take the advice of those who are able to advise. The ordinary aim of the dental student is by hook or crook to get through his course in as short time as possible, and if he can by any circumstance shorten his three years course, he considers it a great gain. Very few students who go over the ordinary curriculm, spend the ordinary time proposing, "I will take further time; I will pursue this course still further." Now and again that sort of student is found, but very seldom. There is a great fault at this point, in the preparation of students, and the success of a man in entering upon a practice, whether city or country, is dependent in a good degree upon the ability he possesses. A young man entering upon his profession with a feeling that he is not prepared as he ought to be, not prepared as he might be, is at a great disadvantage, though he may have all the other things you have referred to; a proper office, a good location, all the matters that have been mentioned. If he is laboring under the impression, "I am inadequately prepared," he is at a great disadvantage preventing his making the success he would make if he could realize, "I am well prepared. I think I can understand that case and the other and the other." The knowledge that he is well prepared is of great help to any young man, and my impression is that about half of our students or more, ought to have a year or a year and a half more in order to make proper success. Another matter which is oftentimes over-looked, neglected or does not receive attention as it ought, is that of association. Meeting with those who are to be competitors. Who are in the same profession. Those in the same vicinity, the same town, the same locality. It is a great matter for a young man starting in a town or city, to have the good will of those who are going to work with him in the same profession. Why is it that there is not a common feeling and sympathy in a common work as in other things? Men engaged in the same occupation, meeting the same difficulties, should have a common sympathy which binds them together and makes each one stronger than it would be possible for them to be standing apart. So it seems that the association of the young man with those about him, is a great factor in his success. Let him meet every dentist in his own town and vicinity. Get this good will, and feel that he is to be a help to him instead of an antagonist. It is a great thing for a young man to have a neighbor who can say, "he is a splendid young man. He will do everything well." A young man ought to pay largely for an opportunity or an advantage of that kind. It is very common however, to go to an office, and if not kindly received, though perhaps that man did not have half an hour to give to conversation, he will say, "I was not kindly received when I went there. I am not going again." A young man should make it a point to secure that object. To secure the good will and approbation of his fellow practitioners; especially those whose esteem is worth anything at all, and put himself in a good position with them. And no right-minded practitioner who has been engaged in the practice, will fail to extend the right kind of spirit to a young man who is earnestly, honestly seeking to enter the profession. How very kindly we sometimes hear persons speak of their first intercourse with an old practitioner. I have often heard that of Dr. Metcalf; that he received them so kindly. Another young man will say, "I called on Dr. J. and he bluffed me. He wouldn't show me anything, and I thought this was not a good place, and that I better go somewhere else." Sometimes that kind of an exhibition is made from a proper cause. Another time a young man would say, "I called upon such a one. He bluffed me. I called again and he was a little kinder. Now we are warm friends." Suppose he had not gone again and had not received the benefit of that friendship—there is a unity of interest that ought to exist between beginners and old practitioners. Each has a duty to perform that ought to be scrupulously attended to. Both will be helped by the proper course in this respect.

Another thing that would be very helpful would be to cultivate the acquaintance of physicians, and be able to communicate with them in an intelligent way on medical subjects. The dental student understands the fundamental principals of medicine, and he should be able to converse upon them. The young man should be well prepared for his work and able to do all he professes to do, and he will then command the respect of everyone. If he is not able to do so, he will have the distrust of all those who learn the fact. It will ruin the confidence necessary to the success of a young man. Cultivate the acquaintance of real honest, earnest medical men and steer clear of quacks. A dentist ought in a good degree to be an educated man. A scientific man. The scientific questions of the day are abroad for everyone's eyes. A good dentist should be able to converse upon them. He is at a disadvantage if he is not able to talk with scientific men and educators. The subject of education is one of interest to everyone in all our communities. Hardly a household anywhere but that the subject of education is an important one there, with some member, at least. If one is lacking in this respect, he is at a disadvantage. If he can talk intelligently helpfully to these persons; if he can talk intelligently to physicians on subjects of interest to them, he may be of service to the physician and they may be mutually helped if they are so disposed. There is a number of points yet that have not been covered by the papers or discussion.

Dr. Hall: There is one thing that begins farther back than anything that has been mentioned. That is in regard to the advice to young men in regard to taking up the practice of dentistry. They should not be encouraged to take up the study of dentistry until fully equipped with a good high school education at least. Take a collegiate course if practicable, and so prepare himself more thoroughly. Then he will be better able to select his position, and have a better standing in the community.

Dr. Fowler: It has been my experience that one of the most helpful things a young man can do is to locate across the street from the best dentist in town. When I left college, I located in a small town across the street from the best dentist there. I knew if my work was to stand comparsion with his, it must be the best that I could do, had I located near a poor dentist, and known that I could easily do better than he, I should not have made so much effort to do my best.

Dr. Lowry: After all has been said and done, a man must not aim to ape anybody else, for he cannot reach success by following someone else. If he succeeds, he must follow out his own character. He must put as much individuality into his work as he possesses. One individual attracts a certain class of people different from another. If you ape another person, you will fail to attract those people whom you would naturally. Let us be ourselves, and at our best all the time. The dentist can least afford to be at a disadvantage of any man living. He must have a good eye, a steady nerve, must be a skillful mechanic. There is no other profession that requires one to be so much of a natural mechanic. That must be developed. If you observe these things and what has been said in the papers and in the discussion, and you have any natural aptitude for the practice of dentistry, you cannot fail to succeed.

Dr. Wood: I have made a practice of keeping a record of all work that I do. When I make an examination, I mark it down on a card which has a diagram on it, and on finishing furnish one to the patient so that they know exactly what I charge for. I have found it very satisfactory.

Dr. Douglas: I urge my patients to come often, as often as once in three months so that I can look over their teeth and make any suggestions necessary. Another thing I think we should do is to make an operation appear as light as possible. I don't think there is anything gained by making out that it is a great miracle, which no one but a dentist knows anything about, and use such extravagant terms which no one can comprehend. Make it simple to them. While they cannot do the mechanical part, they may be made to understand it. I try to make them understand the principles. I believe the better our patients understand these things, the more dentistry there is to do.

---

The Committee of Arrangements for the American Medical Association of 1898 has been called together to take preliminary action for the great gathering which will be held on June 6, 1898.

